# Quality of Life and Neutropenia in Patients with Early Stage Breast Cancer: A Randomized Pilot Study Comparing Additional Treatment with Mistletoe Extract to Chemotherapy Alone

**DOI:** 10.4137/bcbcr.s2905

**Published:** 2009-07-06

**Authors:** Wilfried Tröger, Svetlana Jezdić, Zdravko Ždrale, Nevena Tišma, Harald J. Hamre, Miodrag Matijašević

**Affiliations:** 1 Clinical Research Dr. Tröger; 2 Institute of Oncology and Radiology of Serbia (Belgrade); 3 Institute for Applied Epistemology and Medical Methodology

**Keywords:** mistletoe thereapy, breast cancer, randomized clinical trial, quality of life, neutropenia

## Abstract

**Background:**

Chemotherapy for breast cancer often deteriorates quality of life, augments fatigue, and induces neutropenia. Mistletoe preparations are frequently used by cancer patients in Central Europe. Physicians have reported better quality of life in breast cancer patients additionally treated with mistletoe preparations during chemotherapy. Mistletoe preparations also have immunostimulant properties and might therefore have protective effects against chemotherapy-induced neutropenia.

**Patients and Methods:**

We conducted a prospective randomized open label pilot study with 95 patients randomized into three groups. Two groups received Iscador® M special (IMS) or a different mistletoe preparation, respectively, additionally to chemotherapy with six cycles of cyclophosphamide, adriamycin, and 5-fluoro-uracil (CAF). A control group received CAF with no additional therapy. Here we report the comparison IMS (n = 30) vs. control (n = 31). Quality of life including fatigue was assessed with the European Organization for Research and Treatment of Cancer Quality of Life Questionnaire (EORTC-QLQ-C30). Neutropenia was defined as neutrophil counts <1,000/μl and assessed at baseline and one day before each CAF cycle.

**Results:**

In the descriptive analysis all 15 scores of the EORTC-QLQ-C30 showed better quality of life in the IMS group compared to the control group. In 12 scores the differences were significant (p < 0.02) and nine scores showed a clinically relevant and significant difference of at least 5 points. Neutropenia occurred in 3/30 IMS patients and in 8/31 control patients (p = 0.182).

**Conclusions:**

This pilot study showed an improvement of quality of life by treating breast cancer patients with IMS additionally to CAF. CAF-induced neutropenia showed a trend to lower frequency in the IMS group.

## Introduction

### Background

Quality of life is frequently deteriorated during and after chemotherapy for cancer.^1^ Besides nausea, emesis and pain, fatigue is a frequent complaint, reported by 70%–100% of the patients.^2^ Recent research has shown that fatigue lasts for months after the last cycle of chemotherapy.^2^ Three types of fatigue are distinguished: general, physical, and mental; the latter being related to reduced activity, depression, anxiety, and mood disorders.^3^,^4^ Fatigue, insomnia and daytime sleepiness are augmented during and after chemotherapy with cyclophosphamide and 5-FU combined with methotrexate (CMF) or anthracyclines (CAF).^5^ One can conclude that fatigue is one of the major concerns for patients with cancer.^6^,^7^

### Mistletoe therapy

Physicians have reported better quality of life in breast cancer patients additionally treated with mistletoe preparations during chemotherapy, compared to patients receiving chemotherapy alone.^8^–^12^ Mistletoe preparations also have immunostimulant and DNA-protecting properties^13^,^14^ and might therefore have protective effects against chemotherapy-induced neutropenia.^15^

In Central Europe, mistletoe preparations are widely used for adjuvant cancer therapy, especially by breast cancer patients. In Germany, 70% of cancer patients use unconventional treatments, and randomized trials of mistletoe preparations are difficult to conduct because of non-compliance and low recruitment rates due to therapy preferences.^16^–^19^ Mistletoe preparations^10^,^16^ as adjunct to CMF^12^,^16^ or other chemotherapies for breast cancer and other types of cancer^10^ have been examined in a few clinical studies showing a benefit in EORTC-QLQ-C30^16^ and other quality of life scores.^10^,^12^

This randomised clinical trial assessed mistletoe effects during CAF chemotherapy for breast cancer. The aim was to explore whether the EORTC-QLQ-C30 is a suitable instrument to detect differences between patients with early breast cancer receiving CAF with and without additional mistletoe therapy and whether such study is feasible in Serbia, a country where mistletoe preparations are not available.

## Methods

### Objectives

The objectives of this pilot study were to determine the clinical response (quality of life including fatigue) and neutropenia in breast cancer patients during CAF. Our hypotheses were: Breast cancer patients receiving mistletoe preparations during six cycles of consecutive treatment with CAF will show a better quality of life including fatigue, and less neutropenia compared to patients receiving CAF alone.

### Design

We conducted a prospective randomized open label study with equal-size randomization into three groups. All three groups received six cycles of CAF. In addition, one group received Iscador^®^ M special (IMS), another group received a different mistletoe preparation and a control group had no additional therapy. Here we report the comparison IMS vs. control.

### Participants

Breast cancer patients in the stages T_1–3_N_0–2_M_0_ treated at the Institute of Oncology and Radiology, National Cancer Research Centre of Serbia in Belgrade (IORS) and prescribed six consecutive cycles of CAF after surgery were assessed for eligibility.

Additional inclusion criteria were female gender, age ≥18 years, Karnofsky-Index ≥60, leucocytes ≥3‚000/mm^3^, thrombocytes ≥100‚000/mm^3^, serum creatinine ≤2 mg%, serum glutamic oxaloacetic transaminase (SGOT), and serum glutamic pyruvic transaminase (SGPT) ≤2.5 × the upper institutional limits.

Exclusion criteria were pregnancy or lactation, metastases, planned radiation or hormone therapy during six consecutive cycles of CAF, use of immunostimulant or immunosuppressive agents (e.g. corticosteroids) except for nausea and emesis; current use of other investigational agents, clinically relevant physical or mental illness such as serious infections, hepatic, renal or other organ dysfunction or major depression; alcohol abuse, alcoholism, oral or parenteral drug abuse, and methadone treatment.

### Randomization

The chance to be allocated to any of the three groups (IMS, other mistletoe preparation, control) was 1:1:1. For randomization variable block sizes were used. No stratification took place prior to randomization. The randomization sequence was generated by Clinical Research Dr. Tröger (CRDT), using SPSS^®^ (SPSS^®^ 14.0.1, SPSS Inc., Chicago, Ill, USA). Allocation concealment was implemented by using sealed envelopes, prepared by CRDT. Patients were enrolled by investigators at the Outpatient Clinic, IORS, while the sealed randomization envelopes were stored in the Department of Study Coordination, IORS and released consecutively for each enrolled patient.

### Interventions

CAF was administered in six cycles with a three-week interval between each cycle. The scheduled dosage was 500 mg Cyclophosphamide, 50 mg Adriamycin, and 500 mg 5-FU per 1 m^2^ skin surface applied at one day. All patients received antiemetic therapy with a single dose of Ondansetrone chloride 8 mg, Dexamethasone 8 mg, and Ranitidine 50 mg, respectively, administered prior to each CAF cycle.

One dropout patient in the control group received only three CAF cycles, while all other patients received the six scheduled CAF cycles. The applied dose intensities (DI) of Cyclophosphamide, Adriamycin, and 5-FU (DI in mean mg/m^2^ per week; ± standard deviation) were 160.8 ± 5.5 mg, 16.1 ± 0.5 mg, and 160.8 ± 5.5 mg, respectively, in the IMS group and 157.1 ± 15.6 mg, 15.7 ± 1.6 mg, and 157.1 ± 15.6 mg, respectively, in the control group. The results correspond to 99% of planned DI in the IMS group and 96% of planned DI in the control group. No other antineoplastic or immunomodulating therapies were applied during the study.

Patients randomly allocated to additional therapy with IMS received Iscador^®^ M special (IMS; fermented aqueous extract of Viscum album from apple tree, fresh plant material, ratio of plant to extract = 1:5). IMS was manufactured by Weleda AG, Schwäbisch Gmünd, Germany and prepared in 1 ml ampoules for injection, each ampoule containing the fermented extract of 0.01, 0.1, 1, 2, or 5 mg of fresh mistletoe herb, respectively, in isotonic saline solution. IMS was administered by subcutaneous injections of 1 ml IMS into the upper abdominal region three times per week (i.e. Monday, Wednesday, Friday). The patients were instructed to inject IMS themselves. The dosage of IMS was increased stepwise: 2 × 0.01 mg, 2 × 0.1 mg, 11 × 1 mg, 8 × 2 mg, remaining doses 5 mg. Dose-dependent inflammatory reactions at the injection site (redness, swelling, sometimes accompanied by itching) were monitored. If such reactions exceeded 5 cm in diameter, the dosage was decreased or the therapy was paused until the reactions had ceased. An average of 54.1 ± 2.3 injections with altogether 174.80 ± 26.34 mg of IMS per patient were administered in the IMS group.

### Outcomes

Outcomes were quality of life and neutropenia. Quality of life was assessed using the European Organization for Research and Treatment of Cancer Quality of Life Questionnaire (EORTC-QLQ-C30) in the official Serbian translation.^20^ The EORTC-QLQ-C30 has 30 questions and is analysed in 15 scores: six scores for functioning and nine symptom scores (Table 2). The EORTC-QLQ-C30 was documented by the patients at seven visits: before each CAF cycle and three weeks after the 6th CAF cycle. Neutropenia was defined as neutrophil count <1,000/μl in the peripheral blood and assessed one day before each CAF cycle and three weeks after the 6th CAF cycle.

### Assessment of adverse events (AE)

Adverse events were assessed by interviewing the patients and by analysing the laboratory results at each visit. Local reactions to IMS less than 5 cm in diameter were not considered as adverse events.

### Sample size

A sample size of 90 patients (30 per group) was considered to be sufficient for this pilot study.

### Blinding

The study was not placebo controlled because the typical and time-dependent reactions following s.c. injections of mistletoe extracts (reactions at the injection site, increased body temperature, flu-like symptoms) cannot be imitated by a pseudo-placebo.

### Statistical methods

Statistical analysis (SPSS^®^ 14.0) was performed on the intention-to-treat-population. An alternative analysis of the per-protocol sample, excluding one dropout patient in the control group, yielded very similar results (data not shown). All results of the statistical analysis have exclusively hypothesis-generating character.

For each quality of life score (EORTC QLQ-C30), the mean change from baseline during follow-up in each group was compared between the IMS group, the group with the other mistletoe preparation and the control group, using nonparametric marginal models according to Brunner^21^ with therapy as whole-plot factor and time as sub-plot factor and a possible interaction between these two factors (results not shown). As a sensitivity assessment, a parametric covariance pattern model was also applied and found to qualitatively concur with the nonparametric results. For a better representability, the estimates of this parametric model will be shown. Post-hoc analyses of differences between the IMS group and control group were performed using the Dunnett T3-Test.

Neutropenia was assessed by measuring the number of patients with neutrophils <1,000/μl during the study. For neutropenia, the group difference between IMS group and control group was analysed by Fisher’s exact test according to the sequential rejective Holm procedure.

### Adherence to regulations

The study was approved by the Ethics Committee of the National Cancer Research Center of Serbia without modifications (date: 3 October 2005) and by the Serbian Drug Agency (date: 01 November 2005). Due to its pilot character, this study was not registered in a public study registry. The study was conducted in compliance with the Declaration of Helsinki, Good Clinical Practice guidelines, and national laws. A patient insurance was provided for all participants. All patients provided signed informed consent prior to inclusion. CRDT was responsible for planning, conduct, monitoring, and analysis of the study. Two audits at CRDT and one at the study site were performed by the two sponsors during the study. No violation of Good Clinical Practice was detected.

## Results

### Recruitment, participant flow, assessment, and numbers analysed

**Figure 1.**
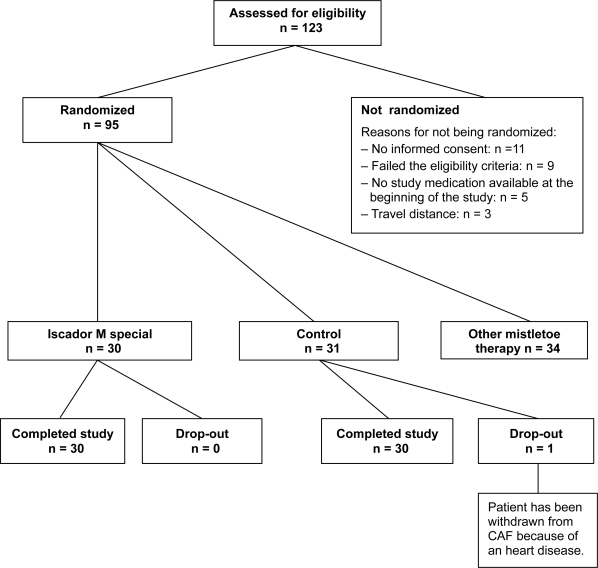
Detailed flow chart of the patient disposition.

From 14 December 2005 to 15 February 2007 a total of 123 breast cancer patients were prescribed CAF and assessed for eligibility at the study centre IORS. 28 patients were not included and 95 patients were included and randomized into 3 groups: CAF and IMS (n = 30), CAF and another mistletoe preparation (n = 34), and CAF without additional therapy (n = 31). One patient in the control group was withdrawn from further CAF therapy after three cycles of CAF because of heart disease (Fig. 1).

The EORTC-QLQ-C30 was evaluable for 99.5% (209 of 210) of visits in the IMS group, and for 97.2% (211 of 217) of visits in the control group. The neutrophil count was assessed at 99.5% (209 of 210) of visits in the IMS group and 98.2% (213 of 217) of visits in the control group.

### Baseline data of the patient groups

The IMS group and the control group did not differ significantly regarding age, tumour stage, body mass index, physical status, vital signs, previous diseases, EORTC-QLQ-C30 scores (Table 1). No neutropenia was detected at baseline.

### Quality of life during chemotherapy

During chemotherapy with CAF a deterioration of quality of life occurred in all 15 EORTC-QLQ-C30 scores in the control group and in six scores in the IMS group. A clinical relevant deterioration of at least 5 points was found in eight scores of the control group and in two scores in the IMS group (Table 3). The most pronounced deteriorations were observed for nausea/emesis (17.2 score points) and fatigue (8.2) after the 2nd cycle of CAF and insomnia (13.1) and diarrhoea (11.9) after the 3rd cycle.

In the adjusted analyses, mean differences from baseline were compared between the two groups for each EORTC-QLQ-C30 score: All 15 comparisons favoured the IMS group, 12 comparisons showed significant differences with p-values ranging from 0.017 to <0.001. Clinically relevant differences of at least 5 points favouring the IMS group were observed for nine EORTC-QLQ-C30 scores (Table 2).

**Figure 2.**
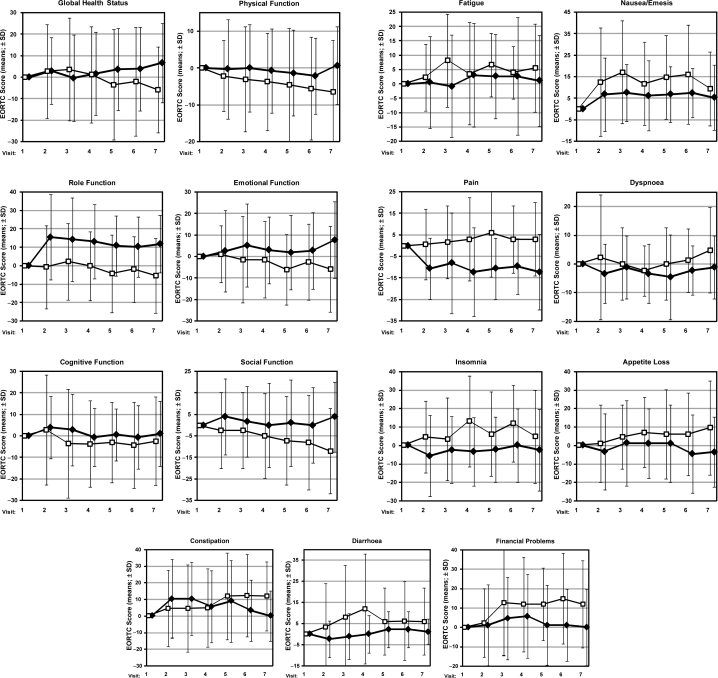
Graphs of the 15 scores of the EORTC-QLQ-C30. The filled markers ♦ represent the IMS group whereas the empty squares □ represent the control group. The mean difference from baseline of the EORTC score (mean; ± SD) is displayed for each visit. In the 6 function scores a higher qualtiy of life is represented by higher values. In the 9 symptom scores a higher quality of life is represented by lower values.

In descriptive analyses, the differences from baseline for each score at each of the six follow-up assessments were compared between the two groups: 86 out of 90 differences favoured the IMS group, while four differences favoured the control group (Fig. 2). Furthermore, the maximum between-group difference for each EORTC-QLQ-C30 score during follow-up was analysed: All 15 differences favoured the IMS group: maximum differences of at least 15 points were found in four scores (Role Function, Social Function, Pain, and Insomnia), 10–15 point differences were found in five scores (Global Health, Emotional function, Constipation, Diarrhoea, Appetite loss), and differences <10 points were found in the remaining six scores.

### Neutropenia during chemotherapy

Neutropenia (<1,000/μl) was detected three times in three different patients of the IMS group and nine times in eight different patients of the control group. The patients with neutropenia are comparable across the groups (Table 3). Odds ratio for the proportion of patients with neutropenia in IMS group vs. control group was 0.32 (95% confidence interval 0.08–1.35). Using Fisher’s exact test according to the sequential rejective Holm procedure, a trend (p = 0.182) towards less neutropenia in the IMS group was found.

### Adverse events related to IMS injections

Localized skin reactions to IMS exceeding 5 cm diameter occurred in six patients. The frequency of these reactions was 0.49% (eight reactions in 1,618 injections). No other adverse events related to IMS occurred.

## Discussion

This randomized pilot study assessed quality of life (EORTC-QLQ-C30) and neutropenia in early breast cancer patients undergoing CAF chemotherapy. Patients receiving IMS in addition to CAF had significantly better quality of life and showed a trend towards less neutropenia, compared to patients receiving CAF alone. IMS therapy was well tolerated.

Strengths of this study include a high recruitment rate, detailed assessments of therapy implementation, high therapy compliance, and very low dropout rates. Since 83% of eligible patients were included and randomized, the results of this study would seem to be generalizable to breast cancer patients (T_1–3_N_0–2_M_0_) receiving CAF without any other concomitant therapies.

Due to the open-label design, the study cannot distinguish between direct drug effects on quality of life and possible indirect effects from therapy expectations, therapy administration etc. in the IMS group. This is a general limitation to all mistletoe studies: mistletoe injections frequently cause local reactions at the injection site, elevated body temperature and flu-like symptoms. These symptoms cease within hours or days after exposure and may recur on repeated injections. Therefore, patients and physicians can easily guess if mistletoe extracts are administered. This was confirmed in a double-blind study, where all patients in the mistletoe group as well as their physicians were unblinded.^16^ A pseudo-placebo causing the same combination of time-limited symptoms—without specific effects on quality of life—has not yet been constructed.

Generally, medication trials are blinded to separate pharmacological effects from placebo effects. However, the lack of blinding may not necessarily have had relevant effects on the results of this study: An updated Cochrane review of randomised trials comparing placebo to no treatment found no significant placebo effects on eight out of ten evaluable indications, small effects on self-reported pain and moderate effects on phobia. Even these effects might have been confounded by biases.^22^–^24^

This study was designed as a pilot study, and the limited sample size of 30 patients per group does not allow for hypothesis confirmation. Nevertheless significant differences in 12 of 15 EORTC-QLQ-C30 scores favouring the IMS group were found. Nine of these scores showed a clinically relevant difference of at least 5 points. The latter scores include pain, nausea, emesis and insomnia, which are highly relevant symptoms in patients during chemotherapy with CAF.

An important problem which may occur during chemotherapy with CAF is neutropenia. Neutropenia is the limiting factor for a continuing chemotherapy and may be harmful to the patients. This study is the first randomized trial providing data on the incidence of CAF-induced neutropenia during additive therapy with IMS. A trend towards less neutropenia in the IMS group was found. This should be further investigated in confirmatory studies with adequate sample size. It is recommended to include measurements of neutrophils on day 7, 9 and day 11 after chemotherapy in order to assess the nadir of the neutropenia during CAF therapy.

Future studies of mistletoe effects on quality of life in cancer patients undergoing chemotherapy should also include specific instruments to assess cancer-related fatigue and pain. In our study the effects of IMS on quality of life increased over time with the largest effects observed at the last follow-up visit. A longer follow-up period of 6–12 months might show additional long term benefits of IMS therapy.

## Conclusions

In this randomized pilot study, additional treatment with mistletoe therapy (Iscador^®^ M special) improved quality of life and showed a trend towards reduction of neutropenia in patients with early breast cancer receiving CAF chemotherapy. These promising results should be confirmed in a larger study.

## Figures and Tables

**Table 1.** Baseline status.

	**Group**		**p values**
	**IMS (n = 30)**	**Control (n = 31)**	
Age in years (mean, ±SD; t-test)	48.4 ± 7.5	50.8 ± 8.0	0.228
Tumour status (n; Chi square test)			
Tumour classification			0.879
T1	7	9	
T2	21	19	
T3	1	2	
Tx	1	1	
Pos. lymph nodes			0.343
N0	10	16	
N1	19	14	
N2	1	1	
Tumour grade			0.963
G1	1	1	
G2	24	24	
G3	5	6	
Menopause status (n; Kruskal-Wallis test)			0.349
Premenopausal	17	13	
Perimenopausal	2	1	
Postmenopausal	11	17	
Receptor status estrogen			0.714
+	20	17	
−	8	12	
Unknown	2	2	
Receptor status progesteron			1.000
+	18	19	
−	10	10	
Unknown	2	2	
Body Mass Index (mean, ±SD; t-test)	27.0 ± 6.3	25.6 ± 4.7	0.709
Karnofsky-Index (mean, ±SD)	100 ± 0.0	100 ± 0.0	
Abnormal findings on physical examination (n)	0	0	
Vital signs (mean, ±SD; t-test)			
Blood pressure systolic mm(Hg)	130.0 ± 20.0	132.2 ± 19.0	0.661
Blood pressure diastolic mm(Hg)	81.7 ± 12.5	83.6 ± 13.6	0.568
Pulse (/m)	82.3 ± 13.4	77.1 ± 10.1	0.094
Temperature (°C)	36.5 ± 0.1	36.6 ± 0.1	0.268
Previous diseases (n)	0	0	
Primary endpoints			
Neutropenia (n)	0	0	
EORTC-QLQ-C30 (mean, ±SD; Mann-Whitney U-test)			
Global health status	67.8 ± 20.4	68.5 ± 18.3	0.868
Physical functioning	86.0 ± 14.2	86.0 ± 12.4	0.852
Role functioning	65.5 ± 20.9	73.0 ± 16.9	0.143
Emotional functioning	75.8 ± 20.1	74.1 ± 18.1	0.623
Cognitive functioning	85.0 ± 20.7	79.3 ± 23.4	0.267
Social functioning	76.7 ± 19.4	80.5 ± 18.9	0.424
Fatigue	22.6 ± 17.2	25.7 ± 19.5	0.593
Nausea and vomiting	3.4 ± 9.3	2.9 ± 7.8	0.959
Pain	21.7 ± 21.5	16.1 ± 20.6	0.210
Dyspnoea	4.6 ± 14.7	3.4 ± 10.3	0.965
Insomnia	16.8 ± 21.1	23.0 ± 31.0	0.557
Appetite loss	12.6 ± 22.6	10.3 ± 20.1	0.625
Constipation	7.8 ± 14.3	6.9 ± 13.7	0.808
Diarrhoea	2.2 ± 8.5	2.3 ± 8.6	0.972
Financial difficulties	23.3 ± 23.4	20.7 ± 30.1	0.358

**Table 2.** EORTC QLQ-C30 scores: mean change from baseline.

**EORTC QLQ-C30 score** **Quality of life**	**Mean change from baseline**			**95% Confidence interval**	**p-value**
	**IMS (n = 30)**	**Control (n = 31)**	**Difference**		
Global health status	2.77	−0.15	2.92	−2.15 to 7.98	0.393
Physical functioning	−0.52	−4.08	3.56	0.61 to 6.51	0.014*
Role functioning	12.88	−1.21	14.09	9.02 to 19.16	<0.001*
Emotional functioning	3.73	−2.62	6.35	2.15 to 10.54	0.001*
Cognitive functioning	1.81	−2.28	4.10	−0.33 to 8.53	0.076
Social functioning	2.08	−5.66	7.74	3.37 to 12.12	<0.001*
Fatigue	0.93	5.84	−4.92	−8.78 to −1.05	0.009*
Nausea and vomiting	7.02	14.60	−7.58	−12.25 to −2.91	0.001*
Pain	−9.81	2.66	−12.47	−16.85 to −8.08	<0.001*
Dyspnoea	−2.32	1.38	−3.70	−6.79 to −0.60	0.015*
Insomnia	−2.53	5.72	−8.25	−13.42 to −3.09	0.001*
Appetite loss	−1.35	5.30	−6.64	−12.28 to −1.01	0.017*
Constipation	7.21	8.33	−1.12	−6.53 to 4.29	1.000
Diarrhoea	0.16	6.27	−6.11	−9.47 to −2.75	<0.001*
Financial difficulties	2.10	11.04	−8.94	−14.21 to −3.67	<0.001*

Difference of the mean of all six follow-up assessments from the baseline: better quality of life is indicated by higher values in the first six scores and by lower scores in the following nine symptom scores.

* Significant difference in the post hoc Dunnett test.

**Table 3.** List of patients experiencing a neutropenia during CAF.

**Group**	**ID**	**Visit no.**	**Date (Visit)**	**Age**	**Stage**	**T**	**N**	**M**	**G**	**Leucocytes/nl**	**Neutrophils/nl**
IMS	37	7	20/10/2006	60	2	2	1	0	2	2.9	0.9
	44	7	03/11/2006	44	2	3	1	0	2	1.8	0.4
	60	2	18/09/2006	51	2	1	0	0	2	6.7	0.6
Control	13	3	26/04/2006	32	2	2	1	0	2	2.8	0.9
	33	5	29/08/2006	60	2	2	0	0	2	2.4	0.9
	51	7	08/12/2006	66	2	2	1	0	2	1.3	0.3
	56	7	21/12/2006	53	2	2	1	0	2	3.5	0.9
	62	7	31/01/2007	62	2	1	1	0	2	2.5	0.8
	66	7	23/01/2007	44	1	1	0	0	2	2.4	0.8
	87	6	26/03/2007	45	2	2	1	0	2	3.3	0.3
	90	6	29/03/2007	52	2	1	0	0	3	2.6	0.8
	90	7	19/04/2007	52	2	1	0	0	3	2.7	0.9

Three patients of the IMS group and eight patients of the control group. Experienced a neutropenia (p = 0.182; 2-sided Fishers exact test).

## Abbreviations

CAF: cyclophosphamide; adriamycin, and 5-fluorouracil;
CRDT: Clinical Research Dr. Tröger;
DI: dose intensity;
EORTC-QLQ-C30: European Organization for Research and Treatment of Cancer Quality of Life Questionnaire;
IMS: Iscador^®^ M special;
IORS: Institute of Oncology and Radiology; National Cancer Research Centre of Serbia in Belgrade.
